# Microscopically controlled two-step surgery for head and neck non-melanoma skin cancer: the role of primary resection and planned secondary wound closure

**DOI:** 10.3389/fonc.2026.1819979

**Published:** 2026-04-20

**Authors:** Gerlind Schneider, Emilie Henkenjohann, Katharina Geißler, Thomas Bitter, Orlando Guntinas-Lichius

**Affiliations:** Department of Otorhinolaryngology, Jena University Hospital, Jena, Germany

**Keywords:** basal cell carcinoma, cutaneous squamous cell carcinoma, keratinocyte cancer, recurrence, skin cancer, surgical margins, two-step surgery

## Abstract

**Objective:**

The aim of the study was to analyze the impact of two-step surgery (primary resection and planned secondary wound closure after final histology results) compared to one-step surgery (resection and immediate wound closure) for non-melanoma skin cancer (NMSC).

**Methods:**

A total of 225 patients with NMSC [median age, 77 years; 68% male; 126 basal cell carcinoma (BCC); 99 cutaneous squamous cell carcinoma (cSCC)] treated between 2010 and 2020 were included. Univariate and multivariate analyses were performed with a main focus on the impact of one-step versus two-step surgery.

**Results:**

One-step versus two-step surgery was performed in 53.8% and 46.2% of the patients. The rate of patients with R+ status after primary resection was lower in the group of patients with planned one-step surgery (23.2%) than in the group of patients with planned two-step surgery (42.3%; p < 0.001). The overall final R+ rate was 4.9%. All these patients belonged to the one-step surgery group. The median follow-up time was 17.5 months. A total of 33 patients (14.7%) had a local tumor recurrence during follow-up. The mean time to recurrence after one-step surgery was 105.0 months (CI = 89.7–120.4). After two-step surgery, it was 126.7 months (CI = 118.0–135.5; p < 0.001). Multivariate Cox regression revealed the following independent risk factors for lower recurrence-free survival: cSCC [hazard ratio (HR) = 6.838; CI = 1.446–32.330; p = 0.015), further head and neck skin cancers after index tumor (HR = 6.262; CI = 1.705–23.001; p = 0.006), and one-step surgery instead of two-step surgery (HR = 6.519; CI = 1.245–8.020; p = 0.048). Re-surgery as a major complication due to bleeding or wound infections (Clavien–Dindo classification grade III) was necessary in 6.2% of the cases. The re-surgery rate was not different between a one-step surgery and a two-step surgery (p = 0.294).

**Conclusions:**

Two-step surgery instead of one-step surgery for unselected cases of both BCC and cSCC was associated with a higher rate of complete tumor resections and a lower recurrence rate.

## Introduction

Non-melanoma skin cancer (NMSC; keratinocyte cancer) is composed of basal cell carcinoma (BCC) and cutaneous squamous cell carcinoma (cSCC). NMSC is the most common type of cancer in the USA and the most frequently diagnosed cancer in fair-skinned populations (1, 2). BCC comprises approximately 80%, and cSCC accounts for 20% of non-melanoma skin cancer diagnoses. Recent studies have suggested an increase in the incidence of cSCC compared to stable incidence rates of BCC (3, 4). This may reflect the aging of the population worldwide. Non-melanoma skin cancer most commonly occurs on the head and neck (approximately 50%) (4). BCC is classified as being at low or high risk of recurrence, and cSCC is classified as being at low, high, or very high risk of recurrence. The risk stratification is based on factors like location, size, status as primary vs. recurrent, histopathological features, history of radiation, and immunosuppression (5).

Both BCC and cSCC can be successfully treated using a variety of modalities (1, 2). Non-surgical destructive options include cryosurgery, electrodessication, curettage, chemical peels, light-based therapies, and topical regimens. These options are widely used for low-risk tumors in non-hair-bearing areas on the trunk and extremities, whereas chemical peels can be used to remove superficial tumors and associated sun damage. Radiation therapy is recommended for non-surgical candidates and as adjuvant treatment. Newer treatments, including targeted therapy and immunotherapy, are mainly reserved for advanced and unresectable cases. Overall, surgery remains the mainstay of treatment for many cases and includes excision with postoperative margin assessments using the “bread loaf” method (6). Microscopically controlled surgery or micrographic surgery refers to tissue-sparing surgical excision of a tumor with traceable markings and subsequent complete histopathological evaluation of lateral and deep margins (7, 8). Especially in the facial area, skin-saving surgery has an obvious priority with regard to aesthetic aspects. The Mohs technique is the most commonly used technique (originally as an in-office procedure using local anesthesia) (9). It needs to be performed by a Mohs specialist (typically dermatologists with specialized training) (1, 2). The immediate pathological evaluation allows an in-office excision until the tumor is completely removed, after which the defect is directly repaired. The day-long process can be time-consuming and is more expensive. Therefore, other micrographic surgery techniques have been published, differing in the surgical incision and histological preparation method, frozen section versus use of paraffin-embedded material, and time to response to the surgeon, ensuring the confirmation of complete resection of the tumor in a different way (7, 8, 10). If the microscopically (micrographically) controlled surgery is based on histological findings in the paraffin section, the result is available at the earliest on the day after the excision (8).

In the case of unfavorable localization, large tumors, or the need for flaps for wound closure, it is advisable not to perform immediate wound closure until clear tumor-free incision margins have been demonstrated. Instead, a two-step surgery consisting of primary resection and planned secondary wound closure after final histopathology is advisable. Therefore, we analyzed an unselected series of 225 patients with non-melanoma skin cancer following microscopically controlled surgery between 2010 and 2020 with special focus on the influence of secondary wound closure on the results, surgical complications, and risk of recurrence.

## Methods

### Study design and setting

The institutional ethics committee of the Jena University Hospital, Jena, Germany, approved the study protocol for retrospective data collection. Only anonymized data were analyzed. Therefore, the ethics committee of the Jena University Hospital, Jena, Germany, waived the need for written informed consent. All methods were performed in accordance with the relevant guidelines and regulations. A standardized retrospective observational study was performed in the Department of Otorhinolaryngology, Jena University Hospital, Jena, Germany.

### Study outcome

The primary outcome was whether one-step surgery or two-step surgery for non-melanoma skin cancer would result in a lower recurrence-free survival rate. Only local recurrences were considered. The secondary outcomes of the study were the overall survival rate and the determination of tumor-related and surgery-related parameters influencing the recurrence-free and overall survival rates.

### Patients and parameters

All patients admitted to the outpatient department of the hospital between January 2010 and December 2020 (to allow a sufficient follow-up time) with the ICD 10 code C44 (malignant skin cancers that are not melanoma) were screened. Hence, the index skin cancer was diagnosed at the latest in 2020. This period was chosen to allow a sufficient follow-up time. These were 299 patients. Only patients with BCC or cSCC, always confirmed by histopathology, in the head and neck region, and scheduled for curative surgical resection were included. Therefore, 74 patients had to be excluded. Finally, 225 patients were included. Patients’ charts were analyzed for patients’ characteristics, comorbidity, prior skin cancer history, surgical and reconstruction details, histopathology, adjuvant therapy, and follow-up data.

A curettage before excision was not performed. A frozen section procedure was not performed during surgery. The tumor specimen was excised in one piece with vertical excisional margins. Then, the tumors were stretched onto cork with the surface facing upward, and a marker was set at 12 o’clock, typically representing the most cranial excision point. Finally, the specimens were fixed in 4% formalin. For all tumors, the initial primary resection margins were individually determined by the surgeon. However, the initial primary resection margins were between 1 and 5 mm, depending on the size and the location of the tumor. The decision of the initial safety margin was individually made based on the size of the tumor and the anatomical localization. In the case of one-step surgery, wound closure was performed directly after tumor resection. In the case of two-step surgery, the wound was covered with a daily ointment dressing and an adhesive bandage until the second step of surgery. The type of wound closure was individually determined by the surgeon. The following techniques were used: primary closure, skin graft, local/regional flap, structural grafts (for the nose region), and combinations with prostheses (for the nose and orbital region). Secondary healing without wound closure was applied in one case.

In all cases, micrographic (microscopically controlled) surgery was performed. Therefore, all tumors were examined by experienced histopathologists from the Department of Pathology, Jena University Hospital, Jena, Germany. All specimens were marked in analogy to a clock for topographic orientation, whereby 12 o’clock was defined as the most cranial point in relation to the body axis. All histological examinations were performed on paraffin-embedded sections. The histological examinations followed the three-dimensional histology (“Tübingen”) technique (11). In the case of positive margins (R+), in most cases, revision surgery was performed. Again, the decision of the safety margin was individually made based on the extension of R+ and the anatomical localization. In the case of planned two-step surgery, this meant that the results of the histopathological examination were awaited once again before the wound was finally closed.

Hence, the number of surgeries per patient varied. In the case of planned one-step surgery, most patients underwent one surgery. Some patients underwent revision surgery because of incomplete primary resection (R+). In the case of planned two-step surgery, most patients underwent two surgeries. Some patients with R+ status after the first surgery did not directly receive a wound closure with the revision surgery because the tumor margins were still unclear. These patients underwent re-resection, but again without wound closure. These patients underwent a planned third surgery after final histopathology.

Surgical complications were graded according to the surgical Clavien–Dindo classification (CDC) (12). The CDC score classifies complications into five groups. The CDC grades the complication based on the severity of the required intervention. In the case of several complications, CDC grading was based on the most extensive intervention. The IBM SPSS Statistics software (Version 28.0.0.0) for medical statistics was used to create a database with standardized parameters.

### Statistical analysis

Participants’ characteristics and outcome variables were analyzed using the IBM SPSS Statistics software (Version 28.0.0.0) for medical statistics. Data are presented as mean ± standard deviation (SD) unless otherwise indicated. The chi-square test was used to compare nominal data of two independent subgroups. Fisher’s exact test was used to compare ordinal data of >2 independent subgroups. The Mann–Whitney U-test was used to compare continuous data of two independent subgroups. Binary logistic regression models were generated to determine odds ratios (ORs) and 95% confidence intervals (CIs) for the analysis of associations between patients with BCC and cSCC, and the absence of complications (CDC 0 versus CDC 1+). Patients’ characteristics for regression analysis were derived from those variables that were significant in preliminary univariate analyses (p < 0.05). The Kaplan–Meier statistics and the log-rank test were used to calculate the probability of tumor recurrence (recurrence-free survival) and death (overall survival). Again, parameters that were significant in preliminary univariate analyses (p < 0.05) were included in multivariate analyses. Multivariate Cox regressions were used to detect independent factors associated with a higher probability of tumor recurrence or death [hazard ratio (HR) and 95% CI]. Patients with missing values were omitted from the univariate or multivariate subanalysis of these parameters. These missing values appeared to occur at random. Also, for the HR analyses, the significance level was set to p < 0.05. In general, nominal p-values of two-tailed tests are reported.

## Results

### Study participants and histological characteristics

A total of 225 patients underwent surgery for non-melanoma skin cancer between 2010 and 2020. The first (index) tumor was BCC in 126 patients (56.0%) and cSCC in 99 patients (44.0%). Patients’ characteristics are summarized in **Supplementary Table 1**. A total of 153 male and 72 female participants (female-to-male ratio, 1:2.4) were analyzed. The median age was 77 years (range, 42–97). The three most frequent tumor localizations were the ear (55.1%), the nose (22.7%), and the eye region (7.6%). Of the tumors, 95% were facial non-melanoma skin cancer.

Out of the 126 index BCC cases, most were nodular BCC (32.9% of all non-melanoma skin cancers; **Supplementary Table 2**). T1 BCC was the most frequent BCC T classification with 40% of all cases (75% of the BCC cases). Higher T classification was rare. Out of the 99 index cSCC cases, most were keratinizing cSCC (44.0% of all non-melanoma skin cancers). T1 cSCC was the most frequent cSCC T classification with 18.2% of all cases (41% of the cSCC cases). A total of 13 cSCC patients had cervical metastasis (N+), and six patients had distant metastasis (M+). The median horizontal diameter and vertical diameter for BCC were 8.0 and 2.3 mm, respectively. The median horizontal diameter and vertical diameter for cSCC were 17.0 and 4.0 mm, respectively.

### Resection with immediate wound closure (one-step surgery) versus planned secondary closure (two-step surgery) and surgery complications

Therapy characteristics are summarized in **Table 1**. Immediate wound closure (one-step surgery) and secondary wound closure after obtaining the histopathology result (two-step surgery) were performed in 53.8% and 46.2%, respectively. If secondary closure was performed, the median interval between primary resection and secondary closure was 5 days. The four most frequent techniques for definitive skin closure were, in descending order, as follows: local flap (58.2%), primary closure (24.9%), local flap and skin graft (7.6%), and only skin graft (6.7%). For cSCC, a neck dissection and/or a parotidectomy was performed in 16.9% and 9.3%, respectively. Postoperative radiotherapy (all in cSCC cases but one BCC case) and systemic therapy were applied in 7.6% and 1.3% of the patients, respectively.

**Table 1:** Therapy characteristics.

Parameter	n	%
All, surgery for skin cancer	225	100
Resection status of all tumors after primary resection		
R0	153	68.0
R1	69	30.7
R2	3	1.3
Resection status BCC after primary resection		
R0	78	34.7
R1	46	20.4
R2	2	0.9
Resection status of cSCC after primary resection		
R0	75	33.3
R1	23	10.2
R2	1	0.4
Second resection because of R+		
Yes	62	27.6
No	10	4.4
Third resection because of persisting R+		
Yes	7	3.1
Second/third resection led to R0		
Yes	61	27.1
No	1	0.4
Resection status of all tumors after final resection		
R0	214	95.1
R1	9	4.0
R2	2	0.9
Wound closure		
Immediate wound closure (one-step surgery)	120	53.3
Secondary wound closure (two-step surgery)	104	46.2
Secondary healing without wound closure	1	0.4
Technique of wound closure		
Local flap	131	58.2
Primary closure	56	24.9
Local flap and skin graft	17	7.6
Skin graft	15	6.7
Prosthesis	2	0.9
Granulation	1	0.4
Unknown	3	1.3
Neck dissection		
Yes, all in case of cSCC	38	16.9
No	187	83.1
Parotidectomy		
Yes, all in case of cSCC	21	9.3
No	204	90.7
Radiotherapy		
Yes, nearly all in case of cSCC (1 for BCC)	17	7.6
No	208	92.4
Systemic therapy		
Yes, all in case of cSCC*	3	1.3
No	222	98.7
	Mean ± SD	Median, range
Interval to wound closure, days	4.6 ± 8.4	2, 0–64
Interval to secondary wound closure only, days	8.5 ± 9.9	5, 1–64

BCC, basal cell carcinoma; cSCC, cutaneous squamous cell carcinoma.

*1× cisplatin, 2× cetuximab.

**Figure 1:**
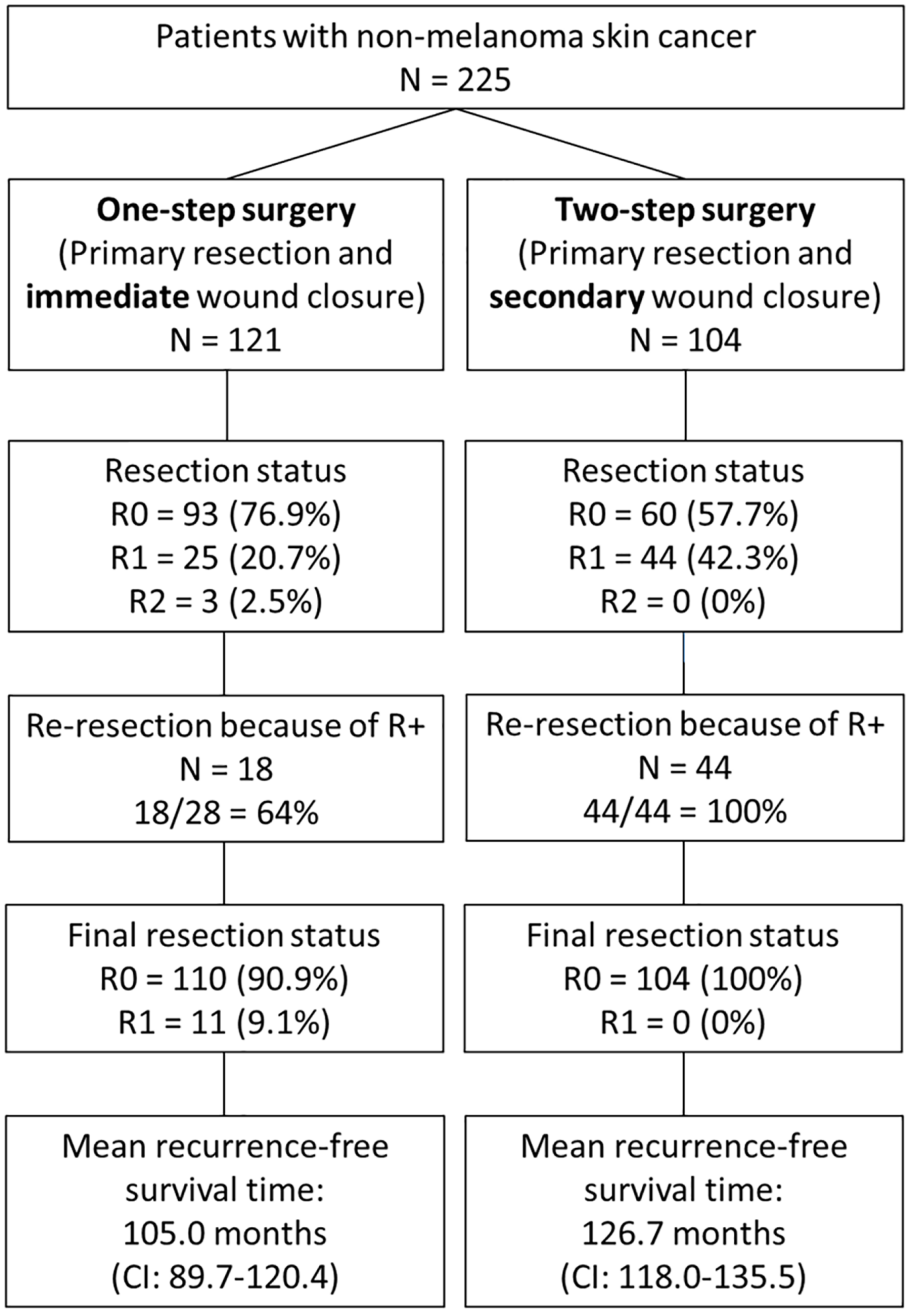
Workflow and tumor margin status R in the one-step surgery group (patients with primary resection with immediate wound closure) versus two-step surgery (planned secondary wound closure).

**Figure 2:**
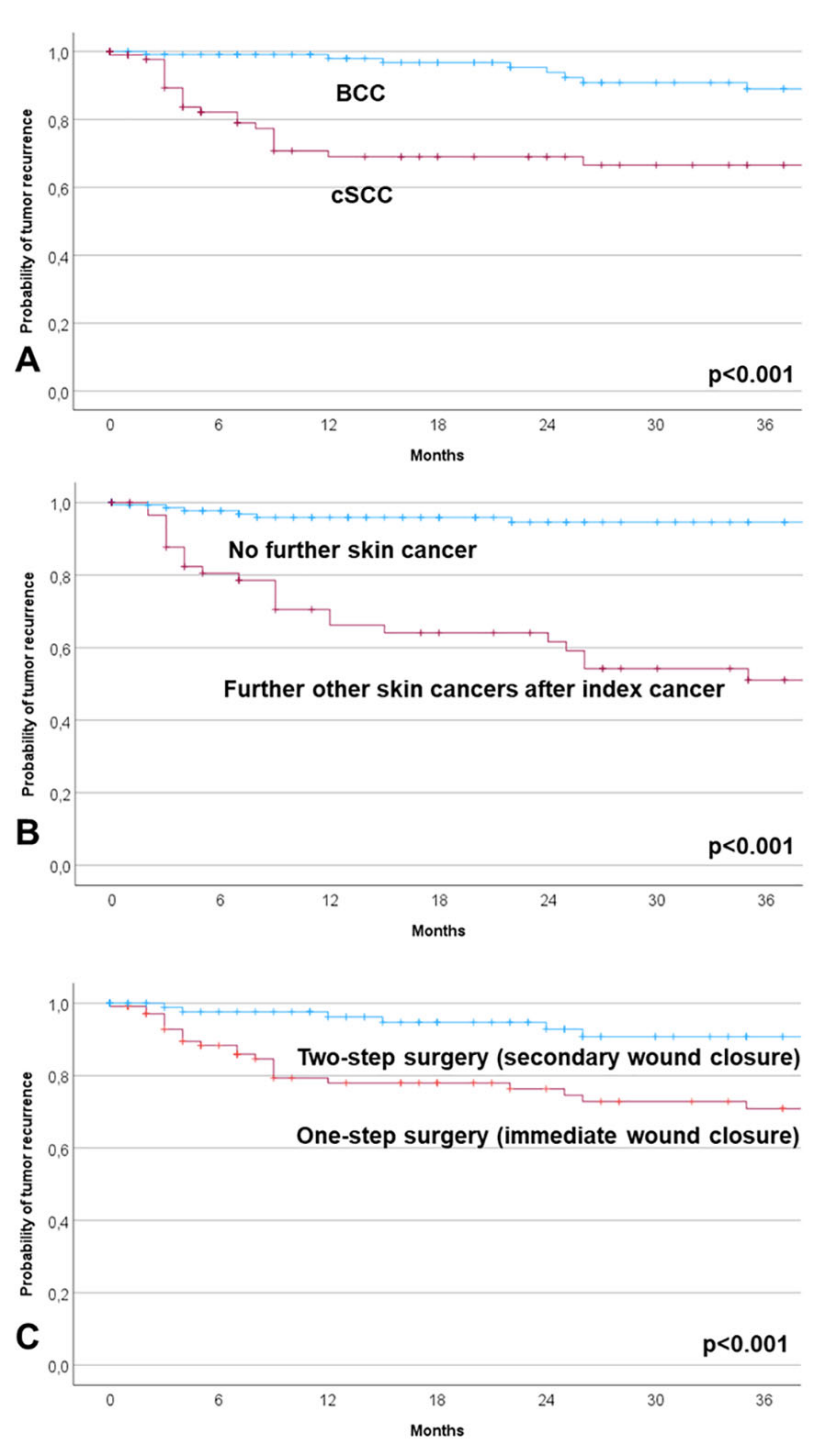
Comparison of one-step surgery (primary resection with immediate wound closure) versus two-step surgery (planned secondary wound closure). **(A)** Two-step surgery was more frequent for basal cell carcinoma (BCC) than for cutaneous squamous cell carcinoma (cSCC; p < 0.001). **(B)** More R+ resections occurred during primary resection in the case of two-step surgery than after one-step surgery (p < 0.001). **(C)** In contrast, more R+ resections occurred after final resection after one-step surgery compared to two-step surgery (p = 0.002).

The workflow and tumor margin status for patients with one-step versus two-step surgery are summarized in **Figure 1**. The resection status of all tumors after primary resection was R0, R1, and R2 in 153 patients (68.0%), 69 patients (30.7%), and 3 patients (1.3%), respectively. The rate of patients with R+ status after primary resection was lower in the group of patients with planned one-step surgery (23.2%) than in the group of patients with planned two-step surgery (42.3%; p < 0.001, **Figure 2**). A re-resection in the group of patients with R+ status (72 patients) was performed in 62 patients (86% of the entire R+ subgroup, 64% of the one-step surgery group, and 100% of the two-step surgery group). The resection status of all tumors after final resection was R0, R1, and R2 in 95.1%, 4.0%, and 0.9%, respectively. The overall final R+ rate was 4.9%. All these patients belonged to the one-step surgery group. Hence, the rate of patients with R+ status after primary resection was now higher in the group of patients with planned one-step surgery (9.2%) than in the group of patients with planned two-step surgery (0%; p < 0.001, **Figure 2**).

The CDC classification of the surgical complication is shown in **Table 2**.** A** total of 127 (56.4%) patients had no postoperative complications. CDC grade I, II, and IIIa/b complications occurred in 18.7%, 17.3%, and 6.2% of the cases, respectively.

**Table 2:** Postoperative complications and follow-up.

Parameter	n	%
All	225	100
Clavien–Dindo classification of complications		
Grade 0 (no complication)	127	56.4
Grade I	42	18.7
Grade II	39	17.3
Grade IIIa	4	1.8
Grade IIIb	10	4.4
Grade IVa	3	1.3
Tumor recurrence (of index skin cancer)		
Yes	33	14.7
No	192	85.3
Death during follow-up		
Yes	50	22.2
No	175	78.2
	Mean ± SD	Median, range
Follow-up, months	27.2 ± 29.9	17.5, 0–151
Follow-up of patients alive, months	34.6 ± 33.6	25, 0.151
Number of recurrences of the index skin cancer	0.2 ± 0.6	0, 0–5

### Univariate and multivariate analyses of factors associated with basal cell carcinoma versus cutaneous squamous cell carcinoma

The univariate comparisons are shown in **Supplementary Table 3**. Male gender, recurrent/progressive tumors as index tumors, localization at other sites such as the nose, localization at the ear, further skin tumors in the further course beyond the index tumor, higher T classification, R+ after final surgery, primary wound closure, neck dissection, radiotherapy, and a larger horizontal tumor diameter were more frequently observed in cSCC compared to BCC (all p < 0.05). In multivariate analysis (**Table 3**), male gender (OR = 2.634, 95% CI = 1.124–6.176; p = 0.026), T4 classification (OR = 7.669, CI = 1.359–43.293; p = 0.021), and a higher horizontal tumor diameter (OR = 1.049, CI = 1.003–1.097; p = 0.038) remained independent factors associated with a higher probability of occurring in cSCC compared to BCC.

**Table 3:** Multivariate binary logistic regression analysis of associations between patients’ and treatment characteristics versus higher probability that the tumor is a cSCC than a BCC*.

Parameter	OR	95% CI Lower	95% CI Upper	p
Gender				
Female	1	Reference		
Male	2.634	1.124	6.176	**0.026**
Index tumor status				
Primary naïve tumor	1	Reference		
Recurrent tumor	0.334	0.107	1.038	0.058
Progressive tumor	NA			
Further head and neck skin cancers after index tumor				
No	1	Reference		
Yes	0.879	0.366	2.115	0.774
Localization on the nose				
No	1	Reference		
Yes	0.591	0.210	1.663	0.319
T classification				
T1	1	Reference		
T2	1.528	0.504	4.630	0.453
T3	3.331	0.856	12.965	0.083
T4	7.669	1.359	43.293	**0.021**
Resection status after final resection				
R0	1	Reference		
R+	0.541	0.076	3.858	0.540
Wound closure				
Immediate wound closure (one-step surgery)	1	Reference		
Secondary wound closure (two-step surgery	0.530	0.233	1.206	0.130
Secondary healing without wound closure**	NA			
Technique of wound closure				
Local flap	1	Reference		
Skin graft	1.603	0.458	5.617	0.461
Primary closure	1.038	0.401	2.686	0.939
Local flap and skin graft	0.329	0.056	1.929	0.218
Prosthesis	NA			
Granulation	NA			
Unknown	NA			
Horizontal tumor diameter, in mm	1.049	1.003	1.097	**0.038**

Significant p-values (p < 0.05) in bold.

NA, not applicable; BCC, basal cell carcinoma; cSCC, cutaneous squamous cell carcinoma.

*Neck dissection, parotidectomy, and radiotherapy were not considered because these treatments were only performed for cSCC.

**For dichotomized statistics, this patient was assigned to the one-step surgery group.

### Univariate and multivariate analyses of factors associated with complications

The results of the univariate analyses for factors associated with relevant complications (CDC grade 1+) are summarized in **Supplementary Table 4**. Distant metastasis (M1), a performance of a neck dissection, and a parotidectomy were the only factors associated with CDC grade 1+ complications (all p < 0.05). In multivariate analysis (**Table 4**), none of these three factors remained as an independent factor associated with a higher risk of CDC grade 1+ complications.

**Table 4:** Multivariate binary logistic regression analysis of associations between patients’ and treatment characteristics for a higher probability of postoperative complications (CDC grade 1+).

Parameter	OR	95% CI Lower	95% CI Upper	p
M classification				0.352
M0	1	Reference		
M1	3.109	0.286	33.848	
Neck dissection				0.271
No	1	Reference		
Yes	1.660	0.673	4.096	
Parotidectomy				0.567
No	1	Reference		
Yes	1.452	0.405	5.204	

### Univariate and multivariate analyses of factors associated with planned secondary wound closure (two-step surgery) versus immediate wound closure (one-step surgery)

All results of the univariate analyses can be found in **Supplementary Table 5**. Two-step surgery was more often used in BCC, recurrent tumors as index tumor, localization on the nose, localization other than the eye region, further head and neck skin cancers after index tumor, lower T classification, N0, M0, lower Union internationale contre le cancer (UICC) stage, R1 after first resection, R0 after final resection, no primary wound closure as technique for closure, no neck dissection, no radiotherapy, and for tumors with a lower horizontal tumor diameter (all p < 0.05; for role of R status cf. **Figure 2**). In multivariate analysis, only including the pre-surgical factors (i.e., patients’ and tumor characteristics; **Table 5**), a BCC (OR = 2.341; CI = 1.258–4.357; p = 0.007) and tumor localized on the nose (OR = 2.332; CI = 1.137–4.784; p = 0.021) were independent factors associated with two-step surgery. There was a trend to a lower probability of two-step surgery in the case of UICC stage IV tumors (OR = 0.336; CI = 0.113–1.002; p = 0.050).

**Table 5:** Multivariate binary logistic regression analysis of associations between patients’ and treatment characteristics versus higher probability of two-step surgery (planned secondary wound closure).

Parameter	OR	95% CI Lower	95% CI Upper	p
Index non-melanoma skin cancer				
Cutaneous squamous cell carcinoma (cSCC)	1	Reference		
Basal cell carcinoma (BCC)	2.341	1.258	4.357	**0.007**
Index tumor status				
Primary naïve tumor	1	Reference		
Progressive tumor	NA			
Recurrent tumor	1.177	0.552	2.510	0.672
Localization on the nose				
No	1	Reference		
Yes	2.332	1.137	4.784	**0.021**
UICC stage				
Stage I	1	Reference		
Stage II	0.701	0.297	1.651	0.416
Stage III	0.535	0.203	1.410	0.206
Stage IV	0.336	0.113	1.002	0.050

Significant p-values (p < 0.05) in bold.

NA, not applicable.

### Univariate and multivariate analyses of recurrence-free survival and overall survival

The median follow-up time was 17.5 months. The median follow-up time of patients without recurrence was 20.0 months. The median follow-up time of patients alive was 25 months. A total of 33 patients (14.7%) had a local tumor recurrence, and 50 patients (22.2%) died during follow-up. The mean interval to recurrence was 120.1 months (CI = 110.3–130.0). The mean interval to death was 108.7 months (CI = 96.8–102.6).

**Figure 3:**
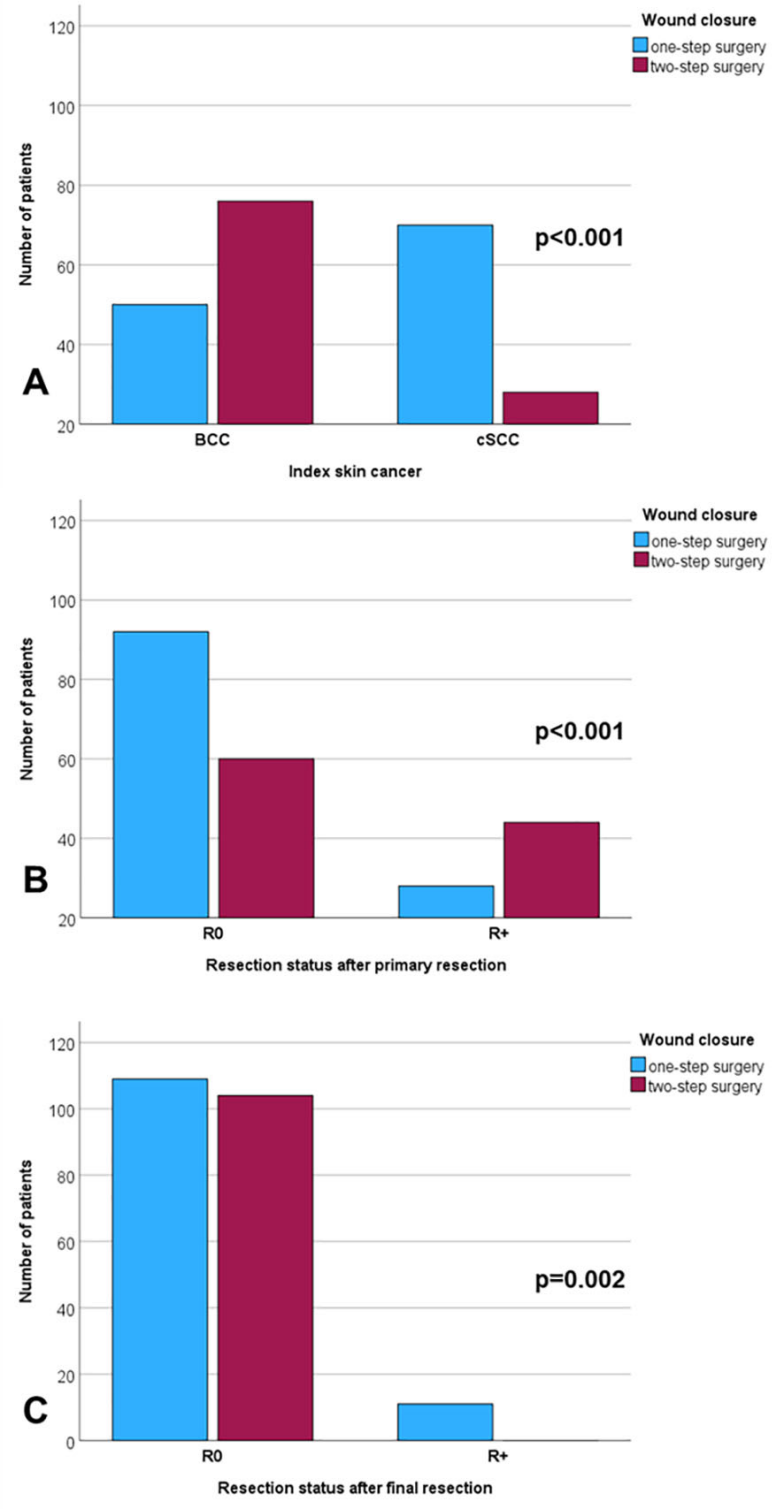
Kaplan–Meier curves for recurrence-free survival. **(A)** Lower probability for cutaneous squamous cell carcinoma (cSCC) than for basal cell carcinoma (BCC; p < 0.001). **(B)** Lower probability patients with further other types of non-melanoma skin cancers after the index cancer compared to patients with a single non-melanoma skin cancer (p < 0.001). **(C)** Lower probability of one-step surgery (patients with immediate wound closure) compared to two-step surgery (patients with planned secondary wound closure; p < 0.001).

The results of all the Kaplan–Meier analyses on recurrence-free survival are shown in **Supplementary Table 6**. The recurrence free survival rate was lower for cSCC than for BCC, lower for progressive/recurrent tumors as index skin cancer, localization not on the nose, localization in the M-zone, further secondary skin cancer during follow-up, history of prior skin cancer, higher T classification, higher N classification, M+, higher UICC stage, higher horizontal tumor diameter, R2 status after first resection, R+ after final resection, immediate wound closure instead of secondary closure, need for neck dissection, parotidectomy, radiotherapy, and patients with complications CDC grade 1+ (all p < 0.05; cf. **Figure 3**). The mean time to recurrence after one-step surgery was 105.0 months (CI = 89.7–120.4); after two-step surgery, it was 126.7 months (CI = 118.0–135.5; p < 0.001; cf. **Figures 1**, **2**). The median time to event, here, i.e., median overall survival, was not reached. Multivariate Cox regression (**Table 6**) revealed the following independent risk factors for lower recurrence-free survival: cSCC (HR = 6.838; CI = 1.446–32.330; p = 0.015), further head and neck skin cancers after index tumor (HR = 6.262; CI = 1.705–23.001; p = 0.006), and immediate wound closure (one-step surgery) instead of planned secondary wound closure (two-step surgery; HR = 6.519; CI = 1.245–8.020; p = 0.048).

**Table 6:** Multivariate Cox regression on influence of patient and tumor characteristics on higher probability of tumor recurrence.

Parameter	HR	95% CI Lower	95% CI Upper	p
Index non-melanoma skin cancer				
Basal cell carcinoma (BCC)	1	Reference		
Cutaneous squamous cell carcinoma (cSCC)	6.838	1.446	32.330	**0.015**
Index tumor status				
Primary naïve tumor	1	Reference		
Progressive tumor	0.309	0.020	4.740	0.399
Recurrent tumor	3.378	0.724	15.749	0.121
Localization on the nose				
No	1	Reference		
Yes	1.145	0.221	5.934	0.872
Localization of index skin cancer				
H-zone	1	Reference		
M-zone	1.930	0.439	8.476	0.384
Further head and neck skin cancers after index tumor				
No	1	Reference		
Yes	6.262	1.705	23.001	**0.006**
History of prior skin cancer				
No	1	Reference		
Yes	4.056	0.738	22.306	0.107
UICC stage				
Stage I	1	Reference		
Stage II	0.244	0.015	4.057	0.325
Stage III	2.545	0.446	14.531	0.293
Stage IV	1.205	0.179	8.095	0.848
Horizontal tumor diameter				
<Median of 12 mm	1	Reference		
≥Median of 12 mm	2.976	0.557	15.900	0.202
Resection status after primary resection				
R0	1	Reference		
R1	2.157	0.320	14.559	0.430
R2	6.660	0.364	121.821	0.201
Resection status after final resection				
R0	1	Reference		
R+	0.225	0.017	2.953	0.256
Wound closure				
Secondary wound closure (two-step surgery)	1	Reference		
Immediate wound closure (one-step surgery)	6.519	1.245	8.020	**0.048**
Technique of wound closure				
Local flap	1	Reference		
Skin graft	NA			
Primary closure	1.491	0.420	5.293	0.537
Local flap and skin graft	3.181	0.215	47.114	0.400
Neck dissection				
No	1	Reference		
Yes	0.363	0.057	2.323	0.284
Parotidectomy				
No	1	Reference		
Yes	1.044	0.199	5.465	0.959
Radiotherapy				
No	1	Reference		
Yes	5.352	0.608	47.140	0.131
CDC				
No (grade 0)	1	Reference		
Yes (grade 1+)	2.522	0.706	9.017	0.155

Significant p-values (p < 0.05) in bold.

HR, hazard ratio; CI, confidence interval; CDC, Clavien–Dindo classification.

The Kaplan–Meier analyses on the overall survival rates are shown in **Supplementary Table 7**. The overall survival rates were lower for cSCC, median age > 77 years, tumor localization in the M-zone, further head and neck skin cancers after index tumor, higher T classification, higher N classification, M1, R2 after first resection, immediate wound closure, neck dissection, parotidectomy, and radiotherapy (all p < 0.05). Multivariate Cox regression analysis (**Table 7**) revealed the following independent risk factors for lower overall survival: age > median of 77 years (HR = 4.031; CI = 1.637–9.924; p = 0.002), tumor in the M-zone (HR = 4.233; CI = 1.608–11.147; p = 0.003), R2 status after first surgery (HR = 7.829; CI = 1.078–56.879; p = 0.042), primary wound closure compared to local flap (HR = 2.586; CI = 1.081–6.189; p = 0.033), and radiotherapy (HR = 3.869; CI = 1.049–14.270; p = 0.042).

**Table 7:** Multivariate Cox regression on influence of patient and tumor characteristics on higher probability of death.

Parameter	HR	95% CI Lower	95% CI Upper	P
Index non-melanoma skin cancer				
Basal cell carcinoma (BCC)	1	Reference		
Cutaneous squamous cell carcinoma (cSCC)	2.064	0.756	5.637	0.157
Age				
≤Median of 77 years	1	Reference		
>M of 77 years	4.031	1.637	9.924	**0.002**
Localization of index skin cancer				
H-zone	1	Reference		
M-zone	4.233	1.608	11.147	**0.003**
Further head and neck skin cancers after index tumor				
No	1	Reference		
Yes	0.976	0.409	2.330	0.957
UICC stage				
Stage I	1	Reference		
Stage II	0.153	0.027	0.887	**0.036**
Stage III	1.167	0.345	3.947	0.804
Stage IV	0.506	0.107	2.401	0.391
Stage unknown	3.254	0.627	16.887	0.160
Resection status after primary resection				
R0	1	Reference		
R1	0.465	0.174	1.242	0.127
R2	7.829	1.078	56.879	**0.042**
Wound closure				
Immediate wound closure	1	Reference		
Secondary wound closure	1.175	0.415	3.323	0.761
Technique of wound closure				
Local flap	1	Reference		
Skin graft	0.580	0.125	2.697	0.487
Primary closure	2.586	1.081	6.189	**0.033**
Local flap and skin graft	NA			
Prosthesis	NA			
Unknown	NA			
Neck dissection				
No	1	Reference		
Yes	1.461	0.417	5.113	0.553
Parotidectomy				
No	1	Reference		
Yes	1.377	0.380	4.988	0.627
Radiotherapy				
No	1	Reference		
Yes	3.869	1.049	14.270	**0.042**

Significant p-values (p < 0.05) in bold.

HR, hazard ratio; CI, confidence interval.

## Discussion

In the present observational study, 225 patients with 356 resected non-melanoma skin cancers received roughly equal proportions of one-step surgery (54%) or two-step surgery (46%). Regardless of the surgical strategy, all cases underwent micrographic (microscopically controlled) surgery based on 3D-histology on paraffin-fixed sections. Hence, none of the presented cases received a standard excision. Nevertheless, both surgical strategy and histological analysis must initially be considered separately from one another. The original Mohs surgery technique, as a popular micrographic surgery strategy in the USA, is based on frozen sections to allow a one-step surgery, as the wound is closed on the day after the tumor has been completely removed, possibly after several subsequent resections (9). The Mohs surgery is costly and resource-intensive. A Mohs surgeon requires specialized training in the interpretation of histopathology slides, and a large surgical space is needed to accommodate several surgeries in parallel, along with the bedside histology laboratory (13). Other micrographic surgery techniques are based on paraffin sections, which also ensure complete resection of the tumor, but can be processed through a standard histology laboratory (10). With this procedure, a two-step surgery is usually mandatory, as the result is not available, like in the present study, on the same day. Several studies have shown that the recurrence rates are lower for micrographic surgery than for conventional surgery, whereby the effect is more pronounced for BCC than for cSCC (cf. study overview in **Supplementary Tables S8**, **S9**; references 23–44). The relapse rate between different micrographic surgery techniques appears to be similar (10, 14).

It is said that standard excision is appropriate for low-risk keratinocyte carcinomas and requires 4–6-mm safety margins (2). Risk stratification is different for BCC and cSCC, but high-risk factors for both include ill-defined borders, histological subtype, perineural invasion, or invasion beyond subcutaneous fat. Since 1) it is often impossible to clearly distinguish between a BCC and a cSCC before surgery, 2) a number of these factors are only known after histological examination, and 3) large safety margins often cannot be maintained, especially in the face, we decided years ago to operate on all non-melanoma skin cancers with a micrographic technique based on formalin-fixed paraffin sections.

The rate of patients with R+ status after primary resection was significantly lower in the group of patients with planned one-step surgery (23.2%) than in the group of patients with planned two-step surgery (42.3%). This has also been reported by others who have also directly compared one-step with two-step surgery (15–17). This is understandable. It is the intention of the planned two-step surgery to be able to deliberately use closer incision margins. Based on the histopathological result, further resection may be performed before definitive wound closure, if necessary. The overall final R+ rate was 4.9%. All these patients belonged to the one-step surgery group, and this speaks in favor of the two-step concept without any patient selection. The literature reports R+ rates of approximately 5%–56% for primary surgery of head and neck BCC and cSCC (cf. study overview in **Supplementary Tables 8**, **S9**). Hence, the R+ rate reported for the present study is satisfactory.

The mean time to recurrence after one-step surgery (105.0 months) was significantly shorter than that after two-step surgery (126.7 months). This corresponded to a 2-year recurrence-free survival rate of 72.8% after one-step surgery compared to 92.8% after two-step surgery. This also speaks in favor of the two-step concept without any patient selection. In the present study, independent risk factors for lower recurrence-free survival were as follows: cSCC compared to BCC, further head and neck skin cancers after the index tumor, and one-step surgery instead of two-step surgery. Other known risk factors include the following: aggressive histopathological subtype or poorly differentiated tumor, tumor extension beyond subcutaneous fat, large caliber nerve invasion, or the presence of a residual tumor or already a recurrent tumor (cf. study overview in **Supplementary Tables 8**, **S9**). Here, again, now related to the risk of recurrence, two-step surgery after a definitive and micrographic histopathology is superior to one-step surgery, which does not wait for final histopathology (2). It does not matter whether a Mohs strategy is used or, as in the present study, the operation is terminated and a second surgery is scheduled.

Re-surgery due to bleeding or wound infection (Clavien–Dindo classification grade III) was the most relevant major postoperative complication and was necessary in 6.2% of the cases. Since skin cancer surgery is mainly performed on older patients, it is particularly important to consider the surgical complications. Nevertheless, we are not aware of any other study using the Clavien–Dindo classification for a systematic analysis of complications after skin cancer surgery (18). In a previous retrospective study of 241 elderly patients, the complication rates (dehiscence, infection, hematoma, and other sequelae) after a one-step and a two-step surgery were 12.5% and 34%, respectively (19). Complications of ambulatory dermatological surgery on 247 successive patients older than 85 years, mainly for head and neck skin cancer, were reported at 7.9% (20). There are even larger series of >1,000 Mohs surgeries (on the entire body), reporting overall complication rates lower than 2% (21).

The present study is not without limitations. Due to the retrospective design, direct causality could not be analyzed. Of course, it would be worthwhile to compare one-step with two-step surgery using the presented approach in a prospective randomized controlled trial. Not all factors were analyzed that may influence the risk of postoperative complications. For instance, frailty as an important factor in elderly patients could not be addressed (22). Furthermore, we were not able to analyze the exact size of the tumor margins in millimeters. This would be of interest for further development of the two-step surgery standard. Finally, both BCC and cSCC can be stratified into low-risk or high-risk for recurrence mainly based on location, size, status as primary vs. recurrent, inflammatory process, patient immunosuppression, histopathological features, and history of radiation or previous treatment (2). Due to the retrospective design, we were not able to stratify the tumors like this because relevant parameters were too often missing. Finally, a Cox regression may not be robust when the number of events is low (only 33 patients experienced a tumor recurrence). In such a case, the regression is prone to overfitting and may overestimate effects. This must be taken into account when interpreting the results of the Cox regression.

In conclusion, the presented unselected and representative series of surgeries on head and neck non-melanoma skin cancer shows that a two-step surgery approach consisting of primary resection without wound closure, detailed spatial microscopic histopathology, and secondary closure after clear margins have been achieved may have advantages in comparison to one-step surgery consisting of tumor resection and direct wound closure. The two-step strategy seems to lead to a lower rate of R+ resections and a lower risk of tumor recurrence without increasing the probability of postoperative complications.

## Data Availability

The original contributions presented in the study are included in the article/**Supplementary Material**. Further inquiries can be directed to the corresponding author.
