# Supplementary material for: Microscopically controlled two-step surgery for head and neck non-melanoma skin cancer: the role of primary resection and planned secondary wound closure
Source: Front Oncol. 2026 Apr 20;16:1819979. doi: 10.3389/fonc.2026.1819979 (PMC13137160; doi:10.3389/fonc.2026.1819979)
Supplement: Supplementary file 1 [file SupplementaryFile1.docx]

**Supplementary Material**

**Microscopically controlled two-step surgery for head and neck non-melanoma skin cancer: the role of primary resection and planned secondary wound closure**

Gerlind Schneider, Emilie Henkenjohann, Katharina Geißler, Thomas Bitter, Orlando Guntinas-Lichius

**Supplementary Table S1.** Patients’ and tumor characteristics.

**Supplementary Table S2.** Histology and staging details of the index tumors separately for the head and neck basal cell carcinomas (BCC) and the cutaneous squamous cell carcinomas (cSCC)

**Supplementary Table S3.** Comparison of characteristics of patients with head and neck basal cell carcinoma (BCC) versus cutaneous squamous cell carcinoma (cSCC)

**Supplementary Table S4.** Comparison of characteristics of non-melanoma skin cancer patients without versus with postoperative complications due the Clavien-Dindo classification.

**Supplementary Table S5.** Comparison of characteristics of non-melanoma skin cancer patients with one-step surgery (immediate wound closure) versus two-step surgery (planned secondary wound closure after histopathology reporting)

**Supplementary Table S6.** Influence of patient and tumor characteristics on probability of recurrence-free survival.

**Supplementary Table S7.** Influence of patient and tumor characteristics on overall survival.

**Supplementary Table S8.** Comparison of the results of selected literature on head and neck basal cell carcinoma (BCC) surgery since the year 2000.

**Supplementary Table S9.** Comparison of the results of selected literature on head and neck cutaneous squamous cell carcinoma (cSCC) surgery since the year 2000.

**References in the Supplementary Tables**

**Supplementary Table S1**

| **Supplementary Table S1.** Patients’ and tumor characteristics. | | | |
| --- | --- | --- | --- |
| **Parameter** | **n** | **%** |  |
| All | 225 | 100 |  |
| Gender |  |  |  |
| Female | 72 | 32.0 |  |
| Male | 153 | 68.0 |  |
| Immunosuppressive therapy |  |  |  |
| Yes | 33 | 14.7 |  |
| No | 192 | 85.3 |  |
| Smoking |  |  |  |
| Yes | 30 | 13.3 |  |
| No | 195 | 86.7 |  |
| Index non-melanoma skin cancer |  |  |  |
| Basal cell carcinoma (BCC) | 126 | 56.0 |  |
| Cutaneous squamous cell carcinoma (cSCC) | 99 | 44.0 |  |
| Index tumor status |  |  |  |
| Primary naïve tumor | 170 | 75.6 |  |
| Recurrent tumor | 43 | 19.1 |  |
| Progressive tumor | 12 | 5.3 |  |
| Localization of index skin cancer |  |  |  |
| Ear | 124 | 55.1 |  |
| Nose | 51 | 22.7 |  |
| Eye region | 17 | 7.6 |  |
| Cheek | 10 | 4.4 |  |
| Forehead | 5 | 2.2 |  |
| Neck | 5 | 2.2 |  |
| Parotid gland | 5 | 2.2 |  |
| Temple | 3 | 1.3 |  |
| Scalp | 3 | 1.3 |  |
| Perioral area | 2 | 0.9 |  |
| H-zone | 202 | 89.8 |  |
| M-zone | 23 | 10.2 |  |
| Right face | 108 | 48.0 |  |
| Left face | 106 | 47.1 |  |
| Central face | 11 | 4.9 |  |
| Further head and neck skin cancers after index tumor |  |  |  |
| No | 163 | 72.4 |  |
| Yes | 62 | 27.6 |  |
| Always basal cell carcinoma (BCC) | 24 | 10.7 |  |
| Always squamous cell carcinoma (cSCC) | 31 | 13.8 |  |
| Index BCC, later also cSCC | 4 | 1.8 |  |
| Index cSCC, later also BCC | 3 | 1.3 |  |
| History for prior skin cancer |  |  |  |
| Basal cell carcinoma | 61 | 27.1 |  |
| Thereof BCC outside head and neck | 22 | 9.8 |  |
| Squamous cell carcinoma | 45 | 20.0 |  |
| Thereof cSCC outside head and neck | 7 | 3.1 |  |
| Melanoma | 1 | 0.4 |  |
| Merkel cell carcinoma | 1 | 0.4 |  |
| Morbus Bowen | 8 | 3.6 |  |
| Fibroxanthoma | 2 | 0.9 |  |
| Keratoacanthoma | 4 | 1.8 |  |
|  | **Mean±SD** | **Median, range** |  |
| Age, years | 76.0±9.5 | 77; 42-97 |  |
| Number of independent head and neck skin cancers | 1.58±1.4 | 1, 1-13 |  |

SD = standard deviation

**Supplementary Table S2**

| **Supplementary Table S2.** Histology and staging details of the index tumors separately for the head and neck basal cell carcinomas (BCC) and the cutaneous squamous cell carcinomas (cSCC). | | | |
| --- | --- | --- | --- |
| **Parameter** | **n** | **%** |  |
| All | 225 | 100 |  |
| Basal cell carcinoma (BCC) | 126 | 56.0 |  |
| Nodular | 74 | 32.9 |  |
| Mixed histology | 21 | 9.3 |  |
| Sclerosing | 11 | 4.9 |  |
| Superficial | 1 | 0.4 |  |
| Infiltrative | 1 | 0.4 |  |
| Not classified | 18 | 8.0 |  |
| T classification |  |  |  |
| T1 | 90 | 40.0 |  |
| T2 | 15 | 6.7 |  |
| T3 | 10 | 4.4 |  |
| T4 | 6 | 2.7 |  |
| Tx | 5 | 2.2 |  |
| Cutaneous squamous cell carcinoma (cSCC) | 99 | 44.0 |  |
| Keratinizing | 75 | 33.3 |  |
| Non-keratinizing | 8 | 3.6 |  |
| Mixed histology | 4 | 1.8 |  |
| Not classified | 12 | 5.3 |  |
| Grading |  |  |  |
| G1 Well differentiated (Low grade) | 18 | 8.0 |  |
| G2 Moderately differentiated (Intermediate grade) | 51 | 22.7 |  |
| Poorly differentiated (High grade) | 22 | 9.8 |  |
| G1-G2 | 1 | 0.4 |  |
| G2-G3 | 2 | 0.9 |  |
| Unknown | 5 | 2.2 |  |
| T classification |  |  |  |
| T1 | 41 | 18.2 |  |
| T2 | 19 | 8.4 |  |
| T3 | 21 | 9.3 |  |
| T4 | 14 | 6.2 |  |
| Tx | 4 | 1.8 |  |
| N classification |  |  |  |
| N0 | 86 | 38.2 |  |
| N1 | 2 | 0.9 |  |
| N2 | 7 | 3.1 |  |
| N3 | 1 | 0.4 |  |
| Nx | 3 | 1.3 |  |
| M classification |  |  |  |
| M0 | 93 | 41.3 |  |
| M1 | 6 | 2.7 |  |
| UICC stage |  |  |  |
| Stage I | 40 | 17.8 |  |
| Stage II | 15 | 6.7 |  |
| Stage III | 17 | 7.6 |  |
| Stage IV | 24 | 10.7 |  |
| Stage unknown | 3 | 1.3 |  |
|  | **Mean±SD** | **Median, range** |  |
| BCC, horizontal diameter, in mm | 11.1±10.0 | 8.0, 0.3-80.0 |  |
| BCC, vertical diameter, in mm | 3.2±3.0 | 2.3, 0.7-19.0 |  |
| cSCC, horizontal diameter, in mm | 20.2 ±12.7 | 17.0, 0.7-63.0 |  |
| cSCC, vertical diameter, in mm | 4.9 ±3.1 | 4.0, 0.6-12.0 |  |

SD = standard deviation

**Supplementary Table S3**

| **Supplementary Table S3.** Comparison of characteristics of patients with head and neck basal cell carcinoma (BCC) versus cutaneous squamous cell carcinoma (cSCC)- | | | |
| --- | --- | --- | --- |
| **Parameter** | **BCC** | **cSCC** | **p** |
| All | 126 | 99 |  |
| Gender |  |  | **0.005** |
| Male | 76 | 77 |  |
| Female | 50 | 22 |  |
| Immunosuppressive therapy |  |  | 0.089 |
| Yes | 14 | 19 |  |
| No | 112 | 80 |  |
| Smoking |  |  | 0.635 |
| Yes | 18 | 12 |  |
| No | 108 | 87 |  |
| Index tumor status |  |  | **0.002** |
| Primary naïve tumor | 97 | 73 |  |
| Recurrent tumor | 28 | 15 |  |
| Progressive tumor | 1 | 11 |  |
| Localization on the nose |  |  | **<0.001** |
| Yes | 41 | 10 |  |
| No | 85 | 89 |  |
| Localization on the ear |  |  | **0.023** |
| Yes | 61 | 63 |  |
| No | 65 | 36 |  |
| Localization in the eye region |  |  | **0.023** |
| Yes | 14 | 3 |  |
| No | 122 | 96 |  |
| Localization of index skin cancer |  |  | 0.085 |
| H-zone | 117 | 85 |  |
| M-zone | 9 | 14 |  |
| Further head and neck skin cancers after index tumor |  |  | **0.020** |
| Yes | 27 | 35 |  |
| No | 99 | 64 |  |
| History for prior skin cancer |  |  | 0.307 |
| Yes | 76 | 53 |  |
| No | 50 | 46 |  |
| T classification |  |  | **<0.001** |
| T1 | 90 | 41 |  |
| T2 | 15 | 19 |  |
| T3 | 10 | 21 |  |
| T4 | 6 | 14 |  |
| Tx | 5 | 4 |  |
| Resection status after primary resection |  |  | 0.087 |
| R0 | 78 | 75 |  |
| R1 | 46 | 23 |  |
| R2 | 2 | 1 |  |
| Resection status after final resection |  |  | **0.049** |
| R0 | 123 | 91 |  |
| R+ | 3 | 8 |  |
| Wound closure |  |  | **<0.001** |
| Immediate wound closure (one-step surgery) | 50 | 70 |  |
| Secondary wound closure (two-step surgery) | 76 | 28 |  |
| Secondary healing without wound closure* | 0 | 1 |  |
| Technique of wound closure |  |  | **0.016** |
| Local flap | 80 | 51 |  |
| Skin graft | 8 | 7 |  |
| Primary closure | 22 | 34 |  |
| Local flap and skin graft | 14 | 3 |  |
| Prosthesis | 1 | 1 |  |
| Granulation | 0 | 1 |  |
| Unknown | 1 | 3 |  |
| Clavien-Dindo-classification of complications |  |  | 0.611 |
| Yes (Grade 1+) | 53 | 45 |  |
| No (Grade 0) | 73 | 54 |  |
| Neck dissection |  |  | **<0.001** |
| No | 126 | 61 |  |
| Yes | 0 | 38 |  |
| Parotidectomy |  |  | **<0.001** |
| No | 126 | 78 |  |
| Yes | 0 | 21 |  |
| Radiotherapy |  |  | **<0.001** |
| No | 125 | 83 |  |
| Yes | 1 | 16 |  |
|  | **Mean±SD** | **Mean±SD** |  |
| Age, years | 74.2±9.9 | 78.2±8.5 | 0.152 |
| Number of independent head and neck skin cancers | 1.4±1.4 | 1.8±1.5 | 0.067 |
| Horizontal tumor diameter, in mm | 11.1±10.0 | 20.2±12.7 | **0.003** |
| Vertical tumor diameter, in mm | 3.3±3.0 | 4.9±3.1 | 0.151 |
| Interval to wound closure, days | 5.7±9.4 | 3.2±6.6 | 0.067 |
| Interval to secondary wound closure only, days | 8.3±10.4 | 8.8±8.6 | 0.787 |

for dichotomized statistics, this patient was assigned to the one-step surgery group; SD = standard deviation; significant p-values (p<0.05) in bold.

**Supplementary Table S4**

| **Supplementary Table S4.** Comparison of characteristics of non-melanoma skin cancer patients without versus with postoperative complications due the Clavien-Dindo classification. | | | |
| --- | --- | --- | --- |
| **Parameter** | **No**  **complications**  **(Grade 0)** | **Complications**  **(Grade 1+)** | **p** |
| All | 127 | 98 |  |
| Index non-melanoma skin cancer |  |  | 0.611 |
| Basal cell carcinoma (BCC) | 73 | 53 |  |
| Squamous cell carcinoma (cSCC) | 54 | 45 |  |
| Gender |  |  | 0.917 |
| Male | 86 | 67 |  |
| Female | 41 | 31 |  |
| Immunosuppressive therapy |  |  | 0.602 |
| Yes | 20 | 13 |  |
| No | 107 | 85 |  |
| Smoking |  |  | 0.225 |
| Yes | 20 | 10 |  |
| No | 107 | 88 |  |
| Index tumor status |  |  | 0.906 |
| Primary naïve tumor | 97 | 73 |  |
| Progressive tumor | 7 | 5 |  |
| Recurrent tumor | 23 | 20 |  |
| Localization on the nose |  |  | 0.302 |
| Yes | 32 | 19 |  |
| No | 95 | 79 |  |
| Localization on the ear |  |  | 0.419 |
| Yes | 67 | 57 |  |
| No | 60 | 41 |  |
| Localization in the eye region |  |  | 0.837 |
| Yes | 10 | 7 |  |
| No | 117 | 91 |  |
| Localization of index skin cancer |  |  | 0.663 |
| H-zone | 115 | 87 |  |
| M-zone | 12 | 11 |  |
| Further head and neck skin cancers after index tumor |  |  | 0.071 |
| Yes | 29 | 33 |  |
| No | 98 | 65 |  |
| History for prior skin cancer |  |  | 0.825 |
| Yes | 55 | 41 |  |
| No | 72 | 57 |  |
| T classification |  |  | 0.431 |
| T1 | 80 | 51 |  |
| T2 | 15 | 19 |  |
| T3 | 17 | 14 |  |
| T4 | 11 | 9 |  |
| Tx | 4 | 5 |  |
| N classification |  |  | 0.625 |
| N0 | 122 | 90 |  |
| N1 | 1 | 1 |  |
| N2 | 3 | 4 |  |
| N3 | 0 | 1 |  |
| Nx | 1 | 2 |  |
| M classification |  |  | **0.046** |
| M0 | 126 | 93 |  |
| M1 | 1 | 5 |  |
| UICC stage |  |  | 0.417 |
| Stage I | 79 | 51 |  |
| Stage II | 15 | 15 |  |
| Stage III | 16 | 11 |  |
| Stage IV | 14 | 16 |  |
| Stage unknown | 3 | 5 |  |
| Resection status after primary resection |  |  | 0.230 |
| R0 | 92 | 61 |  |
| R1 | 34 | 35 |  |
| R2 | 1 | 2 |  |
| Resection status after final resection |  |  | 0.622 |
| R0 | 120 | 94 |  |
| R+ | 7 | 4 |  |
| Wound closure |  |  | 0.294 |
| Immediate wound closure (one-step surgery) | 72 | 48 |  |
| Secondary wound closure (two-step surgery) | 55 | 49 |  |
| Secondary healing without wound closure* | 0 | 1 |  |
| Technique of wound closure |  |  | 0.165 |
| Local flap | 69 | 62 |  |
| Skin graft | 11 | 4 |  |
| Primary closure | 36 | 20 |  |
| Local flap and skin graft | 7 | 10 |  |
| Prosthesis | 1 | 1 |  |
| Granulation | 0 | 1 |  |
| Unknown | 3 | 0 |  |
| Neck dissection |  |  | **0.021** |
| No | 112 | 75 |  |
| Yes | 15 | 23 |  |
| Parotidectomy |  |  | **0.025** |
| No | 120 | 84 |  |
| Yes | 7 | 14 |  |
| Radiotherapy |  |  | 0.067 |
| No | 121 | 87 |  |
| Yes | 6 | 11 |  |
|  | **Mean±SD** | **Mean±SD** |  |
| Age, years | 75.6±10.0 | 76.5±8.9 | 0.332 |
| Number of independent head and neck skin cancers | 1.6±1.7 | 1.5±0.9 | 0.064 |
| Horizontal tumor diameter, in mm | 14.6±12.3 | 15.5±11.7 | 0.636 |
| Vertical tumor diameter, in mm | 3.9±3.4 | 3.9±2.7 | 0.866 |
| Interval to wound closure, days | 4.8±9.6 | 4.3±6.6 | 0.358 |
| Interval to secondary wound closure only, days | 9.5±11.8 | 7.3±7.2 | 0.057 |

for dichotomized statistics, this patient was assigned to the one-step surgery group; SD = standard deviation; significant p-values (p<0.05) in bold

**Supplementary Table S5**

| **Supplementary Table S5.** Comparison of characteristics of non-melanoma skin cancer patients with one-step surgery (immediate wound closure) versus two-step surgery (planned secondary wound closure after histopathology reporting). | | | |
| --- | --- | --- | --- |
| **Parameter** | **One-step surgery**  **Immediate wound closure** | **Two-step surgery**  **Secondary wound closure** | **p** |
| All | 121 | 104 |  |
| Index non-melanoma skin cancer |  |  | **<0.001** |
| Basal cell carcinoma (BCC) | 50 | 76 |  |
| Squamous cell carcinoma (cSCC) | 71 | 28 |  |
| Gender |  |  | 0.147 |
| Male | 87 | 66 |  |
| Female | 34 | 38 |  |
| Immunosuppressive therapy |  |  | 0.743 |
| Yes | 19 | 14 |  |
| No | 102 | 90 |  |
| Smoking |  |  | 0.109 |
| Yes | 12 | 18 |  |
| No | 109 | 86 |  |
| Index tumor status |  |  | **0.006** |
| Primary naïve tumor | 88 | 82 |  |
| Recurrent tumor | 21 | 22 |  |
| Progressive tumor | 12 | 0 |  |
| Localization on the nose |  |  | **<0.001** |
| Yes | 16 | 35 |  |
| No | 105 | 69 |  |
| Localization on the ear |  |  | 0.878 |
| Yes | 67 | 57 |  |
| No | 54 | 47 |  |
| Localization in the eye region |  |  | **0.013** |
| Yes | 14 | 3 |  |
| No | 107 | 101 |  |
| Localization of index skin cancer |  |  | 0.148 |
| H-zone | 105 | 97 |  |
| M-zone | 16 | 7 |  |
| Further head and neck skin cancers after index tumor |  |  | **0.005** |
| Yes | 43 | 19 |  |
| No | 78 | 85 |  |
| History for prior skin cancer |  |  | 0.400 |
| Yes | 55 | 41 |  |
| No | 66 | 63 |  |
| T classification |  |  | **0.008** |
| T1 | 57 | 74 |  |
| T2 | 21 | 13 |  |
| T3 | 23 | 8 |  |
| T4 | 14 | 6 |  |
| Tx | 6 | 3 |  |
| N classification |  |  | **0.040** |
| N0 | 109 | 103 |  |
| N1 | 1 | 1 |  |
| N2 | 7 | 0 |  |
| N3 | 1 | 0 |  |
| Nx | 3 | 0 |  |
| M classification |  |  | **0.021** |
| M0 | 115 | 104 |  |
| M1 | 6 | 0 |  |
| UICC stage |  |  | **0.002** |
| Stage I | 56 | 74 |  |
| Stage II | 17 | 13 |  |
| Stage III | 19 | 8 |  |
| Stage IV | 24 | 6 |  |
| Stage unknown | 5 | 3 |  |
| Resection status after primary resection |  |  | **<0.001** |
| R0 | 92 | 60 |  |
| R1 | 25 | 44 |  |
| R2 | 3 | 0 |  |
| Resection status after final resection |  |  | **0.002** |
| R0 | 109 | 104 |  |
| R+ | 11 | 0 |  |
| Technique of wound closure |  |  | **<0.001** |
| Primary closure | 52 | 4 |  |
| Local flap | 52 | 79 |  |
| Skin graft | 6 | 9 |  |
| Local flap and skin graft | 5 | 12 |  |
| Prosthesis | 2 | 0 |  |
| Unknown | 3 | 0 |  |
| Clavien-Dindo-classification of complications |  |  | 0.284 |
| Yes (Grade 1+) | 48 | 49 |  |
| No (Grade 0) | 72 | 55 |  |
| Neck dissection |  |  | **<0.001** |
| No | 86 | 100 |  |
| Yes | 34 | 4 |  |
| Parotidectomy |  |  | **<0.001** |
| No | 100 | 103 |  |
| Yes | 20 | 1 |  |
| Radiotherapy |  |  | **0.003** |
| No | 105 | 102 |  |
| Yes | 15 | 2 |  |
|  | **Mean±SD** | **Mean±SD** |  |
| Age, years | 77.6±8.6 | 73.9±10.0 | 0.157 |
| Number of independent head and neck skin cancers | 1.7±1.5 | 1.4±1.3 | 0.057 |
| Horizontal tumor diameter, in mm | 17.1±12.4 | 12.0±10.7 | **0.038** |
| Vertical tumor diameter, in mm | 4.4±2.9 | 3.6±3.3 | 0.963 |

*One patient received a resection following by secondary healing without surgical wound closure was assigned to the one-step surgery group; SD = standard deviation; significant p-values (p<0.05) in bold

**Supplementary Table S6**

| **Supplementary Table S6.** Influence of patient and tumor characteristics on probability of recurrence-free survival. | | | |
| --- | --- | --- | --- |
| **Parameter** | **1-year**  **recurrence free rate, %** | **2-year**  **recurrence free rate, %** | **p** |
| All | 86.7 | 84.2 |  |
| Index non-melanoma skin cancer |  |  | **<0.001** |
| Basal cell carcinoma (BCC) | 98.0 | 93.8 |  |
| Squamous cell carcinoma (cSCC) | 69.2 | 69.2 |  |
| Gender |  |  | 0.613 |
| Male | 87.5 | 86.9 |  |
| Female | 98.3 | 86.9 |  |
| Age |  |  | 0.100 |
| ≤median of 77 years | 90.8 | 88.3 |  |
| > median of 77 years | 80.1 | 77.5 |  |
| Immunosuppressive therapy |  |  | 0.981 |
| Yes | 88.0 | 88.0 |  |
| No | 86.5 | 83.5 |  |
| Smoking |  |  | 0.815 |
| Yes | 87.1 | 75.4 |  |
| No | 86.8 | 86.0 |  |
| Index tumor status |  |  | **0.003** |
| Primary naïve tumor | 89.7 | 88.5 |  |
| Progressive tumor | 64.2 | 64.2 |  |
| Recurrent tumor | 70.0 | 70.0 |  |
| Localization on the nose |  |  | **0.028** |
| Yes | 95.1 | 95.1 |  |
| No | 83.6 | 80.3 |  |
| Localization on the ear |  |  | 0.783 |
| Yes | 84.3 | 81.5 |  |
| No | 88.8 | 86.8 |  |
| Localization in the eye region |  |  | 0.677 |
| Yes | NA | 85.7 |  |
| No | 85.2 | 83.4 |  |
| Localization of index skin cancer |  |  | **0.047** |
| H-zone | 87.5 | 84.8 |  |
| M-zone | 83.9 | 43.6 |  |
| Further head and neck skin cancers after index tumor |  |  | **<0.001** |
| Yes | 87.7 | 78.6 |  |
| No | 95.9 | 94.6 |  |
| History for prior skin cancer |  |  | **0.009** |
| Yes | 80.6 | 76.7 |  |
| No | 90.7 | 89.2 |  |
| T classification |  |  | **<0.001** |
| T1 | 94.6 | 93.2 |  |
| T2 | 89.7 | 89.7 |  |
| T3 | 60.0 | 60.0 |  |
| T4 | 67.2 | 67.2 |  |
| Tx | NA |  |  |
| N classification |  |  | **<0.001** |
| N0 | 87.6 | 85.1 |  |
| N1 | NA |  |  |
| N2 | 42.9 | 42.9 |  |
| N3 | NA |  |  |
| Nx | NA |  |  |
| M classification |  |  | **0.015** |
| M0 | 87.2 | 84.6 |  |
| M1 | 62.5 | 62.5 |  |
| UICC stage |  |  | **<0.001** |
| Stage I | 94.6 | 93.1 |  |
| Stage II | 96.0 | 96.0 |  |
| Stage III | 62.3 | 62.3 |  |
| Stage IV | 59.7 | 59.7 |  |
| Stage unknown | 100 | 53.3 |  |
| Horizontal tumor diameter |  |  | **<0.001** |
| <median of 12 mm | 98.4 | 96.4 |  |
| ≥median of 12 mm | 75.9 | 75.9 |  |
| Vertical tumor diameter |  |  | 0.158 |
| <median of 3.5 mm | 95.8 | 95.8 |  |
| > median of 3.5 mm | 91.7 | 91.7 |  |
| Resection status after primary resection |  |  | **0.040** |
| R0 | 86.5 | 85.3 |  |
| R1 | 87.9 | 82.4 |  |
| R2 | 50.0 | 50.0 |  |
| Resection status after final resection |  |  | **0.001** |
| R0 | 87.8 | 85.3 |  |
| R+ | 44.4 | 44.4 |  |
| Wound closure |  |  | **<0.001** |
| Immediate wound closure (one-step surgery) | 77.9 | 72.8 |  |
| Secondary wound closure (two-step surgery) | 96.2 | 92.8 |  |
| Technique of wound closure |  |  | **<0.001** |
| Primary closure | 78.6 | 74.7 |  |
| Local flap | 87.5 | 84.8 |  |
| Skin graft | 100 | 100 |  |
| Local flap and skin graft | 93.3 | 93.3 |  |
| Prosthesis | NA |  |  |
| Granulation | NA |  |  |
| Unknown | NA |  |  |
| Neck dissection |  |  | **<0.001** |
| Yes | 57.8 | 57.8 |  |
| No | 92.1 | 89.2 |  |
| Parotidectomy |  |  | **<0.001** |
| Yes | 39.3 | 39.3 |  |
| No | 90.3 | 87.7 |  |
| Radiotherapy |  |  | **<0.001** |
| Yes | 27.5 | 27.5 |  |
| No | 91.2 | 88.6 |  |
| Clavien-Dindo-classification of complications |  |  | **0.042** |
| Yes (Grade 1+) | 81.5 | 78.0 |  |
| No (Grade 0) | 90.8 | 90.8 |  |

Significant p-values (p<0.05) in bold.

**Supplementary Table S7**

| **Supplementary Table S7.** Influence of patient and tumor characteristics on overall survival. | | | | |
| --- | --- | --- | --- | --- |
| **Parameter** | **1-year**  **survival rate** | **2-year**  **survival rate** | **p** |  |
| All | 92.6 | 86.8 |  |  |
| Index non-melanoma skin cancer |  |  | **<0.001** |  |
| Basal cell carcinoma (BCC) | 98.1 | 94.4 |  |  |
| Squamous cell carcinoma (cSCC) | 84.9 | 76.0 |  |  |
| Gender |  |  | 0.754 |  |
| Male | 89.6 | 87.0 |  |  |
| Female | 94.1 | 86.8 |  |  |
| Age |  |  |  |  |
| ≤median of 77 years | 96.8 | 91.8 | **0.003** |  |
| >median of 77 years | 86.1 | 80.7 |  |  |
| Immunosuppressive therapy |  |  |  |  |
| Yes | 92.3 | 83.1 |  |  |
| No | 92.7 | 87.5 |  |  |
| Smoking |  |  | 0.547 |  |
| Yes | 96.2 | 86.5 |  |  |
| No | 92.0 | 86.8 |  |  |
| Index tumor status |  |  | 0.669 |  |
| Primary naïve tumor | 94.7 | 86.9 |  |  |
| Progressive tumor | 88.8 | 85.1 |  |  |
| Recurrent tumor | 87.5 | 87.5 |  |  |
| Localization on the nose |  |  | 0.198 |  |
| Yes | 97.8 | 89.1 |  |  |
| No | 91.0 | 86.1 |  |  |
| Localization on the ear |  |  | 0.334 |  |
| Yes | 92.9 | 88.1 |  |  |
| No | 92.5 | 85.9 |  |  |
| Localization in the eye region |  |  | 0.097 |  |
| Yes | 100 | 100 |  |  |
| No | 92.7 | 91.3 |  |  |
| Localization of index skin cancer |  |  | **0.008** |  |
| H-zone | 95.6 | 88.3 |  |  |
| M-zone | 75.2 | 75.2 |  |  |
| Further head and neck skin cancers after index tumor |  |  | **0.039** |  |
| Yes | 88.8 | 78.6 |  |  |
| No | 95.5 | 92.1 |  |  |
| History for prior skin cancer |  |  | 0.725 |  |
| Yes | 93.7 | 82.1 |  |  |
| No | 92.3 | 87.8 |  |  |
| T classification |  |  | **0.020** |  |
| T1 | 96.2 | 90.2 |  |  |
| T2 | 96.4 | 96.4 |  |  |
| T3 | 82.3 | 64.7 |  |  |
| T4 | 76.0 | 76.0 |  |  |
| Tx | 100 | 100 |  |  |
| N classification |  |  | **<0.001** |  |
| N0 | 95.3 | 89.2 |  |  |
| N1 | 100 | 100 |  |  |
| N2 | 35.7 | 17.9 |  |  |
| N3 | 100 | 100 |  |  |
| Nx |  |  |  |  |
| M classification |  |  | **0.007** |  |
| M0 | 93.5 | 87.5 |  |  |
| M1 | 53.3 | 53.3 |  |  |
| UICC stage |  |  | **0.022** |  |
| Stage I | 96.2 | 90.1 |  |  |
| Stage II | 100 | 100 |  |  |
| Stage III | 88.2 | 73.5 |  |  |
| Stage IV | 72.8 | 67.6 |  |  |
| Stage unknown | 100 | 100 |  |  |
| Horizontal tumor diameter |  |  | 0.065 |  |
| <median of 12 mm | 96.0 | 91.0 |  |  |
| ≥median of 12 mm | 90.3 | 82.8 |  |  |
| Vertical tumor diameter |  |  | 0.094 |  |
| <median of 3.5 mm | 90.5 | 85.1 |  |  |
| > median of 3.5 mm | 100 | 93.3 |  |  |
| Resection status after primary resection |  |  | **0.009** |  |
| R0 | 92.3 | 84.8 |  |  |
| R1 | 93.2 | 93.2 |  |  |
| R2 | 100 | 50.0 |  |  |
| Resection status after final resection |  |  | 0.456 |  |
| R0 | 92.9 | 87.6 |  |  |
| R+ | 85.7 | 64.3 |  |  |
| Wound closure |  |  | **0.008** |  |
| Immediate wound closure (one-step surgery) | 86.7 | 81.2 |  |  |
| Secondary wound closure (two-step surgery) | 98.9 | 92.5 |  |  |
| Technique of wound closure |  |  | **<0.001** |  |
| Primary closure | 81.4 | 78.5 |  |  |
| Local flap | 96.4 | 87.7 |  |  |
| Skin graft | 90.9 | 90.9 |  |  |
| Local flap and skin graft | 100 | 100 |  |  |
| Neck dissection |  |  | **<0.001** |  |
| Yes | 72.5 | 64.2 |  |  |
| No | 96.7 | 91.5 |  |  |
| Parotidectomy |  |  | **<0.001** |  |
| Yes | 69.7 | 55.8 |  |  |
| No | 94.5 | 89.0 |  |  |
| Radiotherapy |  |  | **<0.001** |  |
| Yes | 64.3 | 41.7 |  |  |
| No | 95.3 | 91.3 |  |  |
| Clavien-Dindo-classification of complications |  |  | 0.991 |  |
| Yes (Grade 1+) | 91.4 | 86.9 |  |  |
| No (Grade 0) | 93.6 | 86.5 |  |  |

Significant p-values (p<0.05) in bold.

**Supplementary Table S8**

| **Supplementary Table S8.** Comparison of the results of selected literature on head and neck basal cell carcinoma (BCC) surgery since the year 2000. | | | | | | | |
| --- | --- | --- | --- | --- | --- | --- | --- |
| **Study** | **Study design** | **Sample size** | **Surgery / histology** | **Follow-up*** | **Recurrence time/ rate** | **Comments** |  |
| Present study | Retrospective | N=126 | One-step; two-step; MCS | Median 17.5 months | mean time to recurrence:  one-step surgery 92.2 months (CI=80.9-103.6); two-step surgery it was to 126.8 months (CI=116.3-137.3) | Time to recurrence longer after two-step surgery |  |
| Lacerda et al. 2024 [^1^](#_ENREF_1) | Meta-analysis | 17 studies; N=4,110 | Conventional surgery; MCS | Selection criterion: >5 years FU | Reduced incidence rate ratio (IRR) for MCS: 0.37 (CI=0.25–0.54) | Recurrence rate lower for MCS than for surgical excision |  |
| Lacerda et al. 2022 [^2^](#_ENREF_2) | Meta-analysis | 18 studies; N=7,362 | Diverse MCS techniques | Selection criterion: >5 years FU | overall recurrence rate:2% (95% CI, 1.0–3.0%) | Recurrence rates between MCS techniques similar |  |
| Fukumoto et al. 2019 [^3^](#_ENREF_3) | Meta-analysis | 14 studies; N=2,524 | Diverse non-surgical therapy and surgery techniques | Selection criterion: >1 year FU | Compared to surgery:  MMS: OR=0.50; CI=0.24–1.03; cryotherapy: OR=4.06, CI=1.65–9.98;  PDT: OR=10.30; CI=4.66–22.74 | Surgery had the lowest odds for recurrence; within surgery techniques; Mohs had the lowest odds for recurrence |  |
| Drucker et al. 2018 [^4^](#_ENREF_4) | Meta-analysis | 45 studies; N=unclear | Diverse non-surgical therapy and surgery techniques | FU < 2 years | Recurrence rates were for excision: 3.8% (CI=1.5-9.5%), Mohs: 3.8% (CI= 0.7%18.2%); curettage/diathermy: 6.9% (CI=0.9-36.6%), radiation: 3.5% (CI=0.7-16.8%). | Excision and Mohs surgery had similar recurrence rates |  |
| Bussu et al 2025 [^5^](#_ENREF_5) | Systematic review | Not separated for BCC/cSCC: 14 studies BCC, 12 studies cSCC; 4 studies both BCC and CSCC; N=6,196; 53% HN | Margin status and local recurrence rate | Range 1 to 279 months | Positive margins: 5-56%  local recurrence rate: 0-20% | Wide variability of rates; no analysis on risk factors |  |
| Kofler et al. 2021 [^6^](#_ENREF_6) | Randomized controlled trial | N=569; tumors ≤30 cm | Serial sections histology versus 3D-histology | Median 4.5 years | Serial sections: re-excisions 21%; recurrence rate: 8.4%;  3D-histology group, re-excisions 39%; recurrence rate 3.5% | Recurrence rate lower for 3D-histology than for serial sections histology |  |
| Van Loo et al. 2014 [^7^](#_ENREF_7) | Randomized controlled trial | N=408 facial BCC; only high risk | Surgical excision versus Mohs MCS | 10-year FU (update of Smeets et al. 2004 [^8^](#_ENREF_8) and Mosterd et al. 2008 [^9^](#_ENREF_9) | Primary BCC, 10-year recurrence probability MCS: 4.4%; surgical excision 12.2%  Recurrent BCC: MCS: 3.9%; surgical excision 13.5% | Recurrence rate lower for MCS than for surgical excision |  |
| Di Maio et al. 2025 [^10^](#_ENREF_10) | Retrospective | N=307 | Positive margins; local recurrence | FU >24 months | Positive margins: 21%  Recurrence rate: 5% | Surgery/histology procedures not clearly described; Higher risk for R+ in nasal BCC |  |
| Kyono et al. 2025 [^11^](#_ENREF_11) | Retrospective | N=48, 60% HN | One-step surgery with surgical excision and 2-mm side margins | Mean 42 months | Recurrence rate: 4.8% | Small sample; results difficult to interpret |  |
| Unlu et al. 2024 [^12^](#_ENREF_12) | Retrospective | N=43 eyelid BCC | One-step versus two-step surgery | Mean 30.9 months for one-step surgery and 37.9 months for two-step surgery | First surgery R+ rate:  One-step surgery: 9%  Two-step surgery 22%  (p=0.241)  No recurrence | Interpretation difficult because of small sample;  higher first surgery R+ rate for two-step surgery |  |
| Iino et al. 2022 [^13^](#_ENREF_13) | Retrospective | N=85 facial BCC | One-step versus two-step surgery | Not presented | R+ after first resection: 1.2%  No recurrence | As follow-up time is unclear, difficult to interpret |  |
| Chouhan et al. 2021 [^14^](#_ENREF_14) | Retrospective | N=307 | Positive margins | Not presented | Positive margins: 7.8% | Half of positive margins: deep margins; most frequent location: temple |  |
| Van Winden et al. 2021 [^15^](#_ENREF_15) | Retrospective | N=89 with 280 BCCs; median age: 83 years | Watchful waiting | Median 9 months | 47% tumors increased in size  Tumor growth was associated with BCC subtype:  OR=3.35; 95%CI, 1.47-7.96; p=0.005);  Estimated tumor diameter increase was 4.46 mm  (80% prediction interval, 1.4-7.46 mm) in 1 year for BCCs containing at least an infiltrative/micronodular component and 1.06 mm(80% prediction interval, −1.79-4.28 mm) for remaining BCCs. | Watchful waiting might be reasonable for asymptomatic nodular or superficial BCCs and a limited life expectancy. |  |
| Dalal et al 2018 [^16^](#_ENREF_16) | Retrospective | N=362 | Positive margins; complications | Not presented | Positive margins: 18%  Complications: 5.8% (for BCC, cSCC, not presented separately) | Most frequent complication: wound infection |  |
| Kuiper et al. 2018 [^17^](#_ENREF_17) | Retrospective | N=1,021 surgeries | MCS | Median 54 months | 5‐year recurrence rate: 3.3%: primary BCC: 2.6%: residual BCC: 5.4%; recurrent BCC: 2.9%  Risk factors: aggressive histopathological subtype, residual BCCs, recurrent BCCs | Higher risk of recurrence in residual or recurrent BCCs |  |
| Goto et al. 2012 [^18^](#_ENREF_18) | Retrospective | N=256; 83% HN | One-step versus two-step surgery | Mean 20 months | Incomplete resection: 8.2%  Recurrence rate: 0.8%  One-step surgery: 0.8%  Two-step surgery: 0% | Recurrence rate very small, therefore not difference between groups |  |
| Boztepe et al. 2004 [^19^](#_ENREF_19) | Retrospective | N=261 | MCS | Mean 5 years | 5-year recurrence rates 3.3% for  primary and 7.3% for recurrent BCC | Higher recurrence rate for recurrent BCC compared to primary BCC |  |

*if given, the median follow-up is presented; BCC = basal cell carcinoma; FU = follow-up; MCS = microscopically controlled two-step surgery; HN = head and neck; OR = odds ration; CI = 95% confidence interval.

**Supplementary Table S9**

| **Supplementary Table S9.** Comparison of the results of selected literature on head and neck cutaneous squamous cell carcinoma (cSCC) surgery since the year 2000. | | | | | | | | |
| --- | --- | --- | --- | --- | --- | --- | --- | --- |
| **Study** | **Study design** | **Sample size** | **Surgery / histology** | | **Follow-up*** | **Recurrence time/ rate** | **Comments** |  |
| Present study | Retrospective | N=99 | One-step; two-step; MCS | | Median 17.5 months | mean time to recurrence:  one-step surgery 78.7 months (CI=67.7-89.6); two-step surgery it was to 89.0 months (CI=67.0-110.9) | Time to recurrence longer after two-step surgery |  |
| Roberts et al. 2025 [^20^](#_ENREF_20) | Meta-analysis | 38 studies; N=1799 with cN0 | Sentinel lymph node biopsy (SLNB), elective dissection (ED), and elective nodal irradiation | | Mean 26.5 months | Overall, 5-year disease-free survival rate: 69.0%;  Overall recurrence SLNB: 8.3%) lower than observation (16.9%) and ED (23.7%) | SLNB strategy for cN0 was associated with lowest recurrence rate |  |
| Lacerda et al. 2024 [^1^](#_ENREF_1) | Meta-analysis | 17 studies; N=1,391 | Conventional surgery; MCS | | Selection criterion: >5 years FU | Incidence rate ratio (IRR):  MCS compared to surgery: IRR=0.57; CI=0.29–1.13 | Recurrence rate equal for MCS and for surgical excision |  |
| Zakhem et al. 2023 [^21^](#_ENREF_21) | Meta-analysis | 129 studies; N=137,449 (HN proportion unclear) | Risk factors for local recurrence | | Not clear | highest risks for local recurrence:  tumor invasion beyond subcutaneous fat: RR=9.1 (CI=2.8-29.2)  MCS had the lowest incidence of nearly all poor outcomes; however, in some results, the 95% CIs overlapped with those of other treatment modalities | Many risk factors listed. |  |
| Sahovaler et al. 2019 [^22^](#_ENREF_22) | Meta-analysis | 21 studies; N=3,534 | Risk factors for OS | | Selection criterion: >1 year FU | Lower OS:  Immunosuppression: HR=2.66 (CI=2.26-3.13; extracapsular spread: HR=1.90 (CI=1.12-3.23); adjuvant radiotherapy: HR=0.45 (CI=0.27-0.78); lymph node ratio: HR=1.91 (CI=1.09-3.35), advanced age (HR=1.03 (CI=1.00-1.07) | Immunosuppression, age, and N histology characteristics relevant risk factors for lower OS |  |
| Thompson et al. 2016 [^23^](#_ENREF_23) | Meta-analysis | 36 studies; N=17,248, but all locations | Not analyzed | | Mean FU, range 30 to 81 months | Risk ratio (RR) for recurrence:  Breslow thickness > 2 mm: RR=9.64; CI=1.30-71.52; invasion beyond subcutaneous fat: RR=7.61; CI=4.17-13.88; Breslow thickness >6 mm: RR: 7.13; CI=3.04-16.72, perineural invasion: RR=4.30; CI=2.80-6.60: Breslow thickness >20 mm: RR=3.22; CI=1.91-5.45; location temple: RR=3.20; CI=1.12-9.15; poor differentiation: RR=2.66; CI=1.72-4.14 | Tumor thickness most relevant risk factor |  |
| Bussu et al 2025 [^5^](#_ENREF_5) | Systematic review | Not separated for BCC/cSCC: 14 studies BCC, 12 studies cSCC; 4 studies both BCC and cSCC; N=6,196; 53% HN | Margin status and local recurrence rate | Range 1 to 279 months | | Positive margins: 5-56%  local recurrence rate: 0-20% | Wide variability of rates; no analysis on risk factors |  |
| Lansbury et al. 2013 [^24^](#_ENREF_24) | Systematic review | 118 studies; N unclear | Recurrence rate | Time unclear | | Pooled estimates of recurrence: cryotherapy: 0.8% (CI=0.1-2%); curettage and electrodesiccation: 1.7% (CI=0.5- 3.4%)), Mohs: 3.0% (CI=2.2-3.9%), standard surgical excision: 5.4% (CI=2.5-9.1%); external radiotherapy: 6.4% (CI=3.0-11.0%) | No clear difference between Mohs and standard surgery |  |
| Porceddu et al. 2025 [^25^](#_ENREF_25) | Secondary analysis of randomized controlled trial [^26^](#_ENREF_26) | N=310 with PORT ± CT | Low-risk versus high-risk group defined as extranodal extension and a nodal size ≥22 mm | Median 60 months | | 5-year DFS:  High-risk: 56% (CI=45-66%); Low-risk: 75% (CI=68%-81%); HR 2.0 (CI=1.3-3.1; p=0.001)  5y-year OS:  High-risk: 59% (CI=46-69%); Low-risk: 85% (CI=79-90%); HR 3.2 (CI=1.9-5.3; p<0.001). | For N+ extranodal extension and size >2 mm are risk factors for recurrence and worse survival |  |
| Ran et al. 2025 [^27^](#_ENREF_27) | Retrospective | N=16,844 invasive cSCC (HN about 60%) | Number of risk factors influence on recurrence | Median 33.6 months | | 4 risk factors: ≥2 cm, poorly differentiated, tumor extension beyond subcutaneous fat, large caliber nerve invasion  Local recurrence risk:  No risk factor: 1.7% (CI=1.5-2.0%; 1 risk factor: 5.0% (CI=4.1-5.9%) ; 2 risk factors: 8.8% (CI= 7.0-11.0%); 3 risk factors: 16.0% (CI=11.0-22.0%); 4 risk factors: 33.0% (CI=19.0-47.0%; p<0.001 | Histology factors influence recurrence risk |  |
| Chouhan et al. 2021 [^14^](#_ENREF_14) | Retrospective | N=199 | Positive margins | Not presented | | Positive margins: 6% | Half of positive margins: deep margins; most frequent location: temple |  |
| Amit et al. 2021 [^28^](#_ENREF_28) | Retrospective | N=1111 with cN0 | Neck dissection versus observation | Median 24-26 months | | 5-year overall survival rate:  Neck dissection: 52%; observation: 63% p= 0.003  5-year disease-free survival rate:  Neck dissections: 73%; observation group: 75%; p=0.429 | No benefit of elective neck dissection, also not in multivariate analysis |  |
| Iino et al. 2022 [^13^](#_ENREF_13) | Retrospective | N=65 facial cSCC | one-step versus two-step surgery | Not presented | | R+ after first resection: 7.7%  No recurrence | As follow-up time is unclear, difficult to interpret |  |
| Dalal et al 2018 [^16^](#_ENREF_16) | Retrospective | N=138 | Positive margins; complications | Not presented | | Positive margins: 7%  Complications: 5.8% (for BCC, cSCC, not presented separately) | Most frequent complication: wound infection |  |
|  |  |  |  |  | |  |  |  |
|  |  |  |  |  | |  |  |  |

*if given, the median follow-up is presented; cSCC = cutaneous squamous cell carcinoma; FU = follow-up; MCS = microscopically controlled two-step surgery; OS = overall survival; DFS= disease-free survival; HN = head and neck; PORT = postoperative radiotherapy; CT = chemotherapy; OR = odds ration; CI = 95% confidence interval.

**References**

1 Lacerda, P. N., Lange, E. P., Luna, N. M., Miot, H. A. & Abbade, L. P. F. Efficacy of micrographic surgery versus conventional excision in reducing recurrence for basal cell carcinoma and squamous cell carcinoma: A systematic review and meta‐analysis. *Journal of the European Academy of Dermatology and Venereology* **38**, 1058-1069 (2024).

2 Lacerda, P. *et al.* Recurrence rate of basal cell carcinoma among different micrographic surgery techniques: systematic review with meta‐analysis. *Journal of the European Academy of Dermatology and Venereology* **36**, 1178-1190 (2022).

3 Fukumoto, T., Fukumoto, R., Oka, M. & Horita, N. Comparing treatments for basal cell carcinoma in terms of long‐term treatment‐failure: a network meta‐analysis. *Journal of the European Academy of Dermatology and Venereology* **33**, 2050-2057 (2019).

4 Drucker, A. M. *et al.* Treatments of primary basal cell carcinoma of the skin: a systematic review and network meta-analysis. *Annals of internal medicine* **169**, 456-466 (2018).

5 Bussu, F. *et al.* Margins in head and neck non-melanoma skin cancer surgery: clinical/pathological criteria and their impact on oncological outcomes and therapeutic choices. A systematic review. *ACTA Otorhinolaryngologica Italica* **45**, S121 (2025).

6 Kofler, L. *et al.* Three‐dimensional histology vs. serial section histology in the treatment of primary basal cell carcinoma: a randomized, prospective, blinded study of 569 tumours. *Journal of the European Academy of Dermatology and Venereology* **35**, 1323-1330 (2021).

7 Van Loo, E. *et al.* Surgical excision versus Mohs’ micrographic surgery for basal cell carcinoma of the face: a randomised clinical trial with 10 year follow-up. *European journal of cancer* **50**, 3011-3020 (2014).

8 Smeets, N. W. *et al.* Surgical excision vs Mohs' micrographic surgery for basal-cell carcinoma of the face: randomised controlled trial. *Lancet* **364**, 1766-1772, doi:10.1016/s0140-6736(04)17399-6 (2004).

9 Mosterd, K. *et al.* Surgical excision versus Mohs' micrographic surgery for primary and recurrent basal-cell carcinoma of the face: a prospective randomised controlled trial with 5-years' follow-up. *The lancet oncology* **9**, 1149-1156, doi:10.1016/S1470-2045(08)70260-2 (2008).

10 Di Maio, P. *et al.* Head and neck cutaneous basal cell carcinoma: a retrospective analysis of tumour features, surgical margins and recurrences. *European Archives of Oto-Rhino-Laryngology*, 1-9 (2025).

11 Kyono, K. *et al.* Outcome of basal cell carcinoma excision with 2 mm surgical margin in Japanese patients: A retrospective study of one-step surgery. *JPRAS open* **43**, 216-226 (2025).

12 Unlu, M., Ozer, F. & Vural, A. A comparison of two surgical techniques for basal cell carcinoma of the eyelid: two-step surgery and one-step surgery. *International ophthalmology* **45**, 6 (2024).

13 Iino, S. *et al.* Retrospective evaluation of the utility of two-step surgery for facial basal cell carcinoma and squamous cell carcinoma. *Frontiers in Surgery* **9**, 915731 (2022).

14 Chouhan, R., Patel, R., Shakib, K. & Mitsimponas, K. Surgical excision of non-melanoma skin cancer: no end in site? *British Journal of Oral and Maxillofacial Surgery* **59**, 1264-1269 (2021).

15 van Winden, M. E. *et al.* Evaluation of watchful waiting and tumor behavior in patients with basal cell carcinoma: an observational cohort study of 280 basal cell carcinomas in 89 patients. *JAMA dermatology* **157**, 1174-1181 (2021).

16 Dalal, A., Ingham, J., Collard, B. & Merrick, G. Review of outcomes of 500 consecutive cases of non-melanoma skin cancer of the head and neck managed in an oral and maxillofacial surgical unit in a District General Hospital. *British Journal of Oral and Maxillofacial Surgery* **56**, 805-809 (2018).

17 Kuiper, E. M., van den Berge, B. A., Spoo, J. R., Kuiper, J. & Terra, J. B. Low recurrence rate of head and neck basal cell carcinoma treated with Mohs micrographic surgery: a retrospective study of 1021 cases. *Clinical Otolaryngology* **43**, 1321-1327 (2018).

18 Goto, M. *et al.* Analysis of 256 cases of basal cell carcinoma after either one‐step or two‐step surgery in a Japanese institution. *The Journal of Dermatology* **39**, 68-71 (2012).

19 Boztepe, G., Hohenleutner, S., Landthaler, M. & Hohenleutner, U. Munich method of micrographic surgery for basal cell carcinomas: 5-year recurrence rates with life-table analysis. *Acta dermato-venereologica* **84**, 218-222 (2004).

20 Roberts, K. A. *et al.* Management of Regional Lymph Nodes in Clinically Node-Negative Cutaneous Squamous Cell Carcinoma of the Head and Neck: A Systematic Review & Meta-Analysis. *Cancers* **17**, 3335 (2025).

21 Zakhem, G. A., Pulavarty, A. N., Carucci, J. & Stevenson, M. L. Association of patient risk factors, tumor characteristics, and treatment modality with poor outcomes in primary cutaneous squamous cell carcinoma: a systematic review and meta-analysis. *JAMA dermatology* **159**, 160-171 (2023).

22 Sahovaler, A. *et al.* Outcomes of cutaneous squamous cell carcinoma in the head and neck region with regional lymph node metastasis: a systematic review and meta-analysis. *JAMA Otolaryngology–Head & Neck Surgery* **145**, 352-360 (2019).

23 Thompson, A. K., Kelley, B. F., Prokop, L. J., Murad, M. H. & Baum, C. L. Risk factors for cutaneous squamous cell carcinoma recurrence, metastasis, and disease-specific death: a systematic review and meta-analysis. *JAMA dermatology* **152**, 419-428 (2016).

24 Lansbury, L., Bath-Hextall, F., Perkins, W., Stanton, W. & Leonardi-Bee, J. Interventions for non-metastatic squamous cell carcinoma of the skin: systematic review and pooled analysis of observational studies. *Bmj* **347** (2013).

25 Porceddu, S. V. *et al.* Prognostic subgroups for disease-free survival with cutaneous squamous cell carcinoma of the head and neck: a secondary analysis of a randomized clinical trial. *JAMA Otolaryngology–Head & Neck Surgery* **151**, 938-945 (2025).

26 Porceddu, S. V. *et al.* Postoperative concurrent chemoradiotherapy versus postoperative radiotherapy in high-risk cutaneous squamous cell carcinoma of the head and neck: the randomized phase III TROG 05.01 trial. *Journal of Clinical Oncology* **36**, 1275-1283 (2018).

27 Ran, N. A. *et al.* Risk factor number and recurrence, metastasis, and disease-related death in cutaneous squamous cell carcinoma. *JAMA dermatology* **161**, 597-604 (2025).

28 Amit, M. *et al.* Elective neck dissection versus observation in patients with head and neck cutaneous squamous cell carcinoma. *Cancer* **127**, 4413-4420 (2021).
